# Tumor Genomics and Liquid Biopsy in Cancer Biology: From Static Snapshots to Dynamic Measurements

**DOI:** 10.3390/biom16030384

**Published:** 2026-03-03

**Authors:** Milena Urbini

**Affiliations:** Biosciences Laboratory, IRCCS Istituto Romagnolo per lo Studio dei Tumori (IRST) “Dino Amadori”, 47014 Meldola, Italy; milena.urbini@irst.emr.it

## 1. Introduction

Cancer is a dynamic and evolving biological system. Tumor genomes change under pressures due to growth, immune and microenvironmental factors, and especially therapy. Tissue biopsy remains essential for diagnosis, histopathology, tumor classification, and treatment selection. However, tissue biopsy provides only a snapshot of a moving target: spatial heterogeneity may separate driver alterations across lesions, and temporal evolution can reshape clonal architecture among diagnosis, treatment, and relapse.

Liquid biopsy, based on the molecular analysis of tumor-derived material in blood and other biofluids, has emerged as a complementary approach to tissue biopsy to better capture the dynamics of cancer biology. By enabling repeated sampling over time, liquid biopsy can broaden the spectrum of tumor-derived information that can be detected and support longitudinal assessment of tumor burden, minimal residual disease, resistance mechanisms, and early malignant changes. This editorial reviews the advances in molecular tumor profiling and biomarker monitoring made possible by liquid biopsy. The analyses of circulating tumor DNA (ctDNA) and other analytes, including circulating tumor cells (CTCs), extracellular vesicles (EVs), tumor-educated platelets (TEPs), circulating RNAs, are covered. How these technologies broaden the spectrum of tumor-derived information that can be detected is outlined, supporting the longitudinal assessment of tumor burden, minimal residual disease, resistance mechanisms, and early malignant changes, emphasizing the biological and practical factors that define clinical and translational utility.

## 2. Liquid Biopsy for Cancer Profiling and Disease Monitoring

Cancer is not a static mass but an evolving ecosystem in which genetically and phenotypically distinct subclones compete, cooperate, and adapt across space and time [1]. Therapy then acts as an intense selective pressure, reducing the presence of sensitive populations, while populations of rare resistant clones expand, modifying the tumor clonal architecture between diagnosis, response, and relapse [2]. In this context, liquid biopsy allows longitudinal studies: repeated sampling across treatment cycles can map how tumors respond, adapt, and re-expand, offering real-time hypotheses about the mechanisms. Moreover, this heterogeneity is not confined to cancer cells; the spatial organization of immune, stromal, and vascular niches can vary sharply within millimeters, and tumor biopsies often represent only a small fraction of the total microenvironment. A tissue biopsy may miss a subclone that is rare locally but expanding systemically; a blood-based signal can, in some contexts, integrate across lesions, providing a complete view of the alterations present.

Liquid biopsy includes the molecular analysis of tumor-derived material in blood and other biofluids. The detection and analysis of ctDNA are the most broadly used approaches for tumor detection and genomic profiling [3,4,5].

ctDNA levels reflect a composite of processes: cell death, tumor vascularity, anatomical site, therapy-induced cytotoxicity, and clearance kinetics. It has been widely demonstrated that shifts in ctDNA composition provide direct evidence of clonal selection—sometimes earlier than radiographic change—revealing resistance as a measurable evolutionary trajectory rather than a retrospective endpoint [6,7,8,9]. In addition to ctDNA, the field of liquid biopsy encompasses the analysis of several other analytes, including CTCs, EVs, TEPs, and circulating RNAs [10].

However, the analysis performed through liquid biopsy has some limitations. Specifically, some tumors types (specific primary sites or early-stage disease) may have limited ctDNA shedding. Thus the absence of detectable ctDNA is not always synonymous with the absence of disease but with the reach of the limit of detection of the method used. Analyses of tumor tissue should be considered in these cases [11,12]. Despite this, in some situations, the absence of detectable ctDNA could still be useful as an indicator of lower disease burden and may thus support prognostic stratification [13]. Another important limitation is the fact that the tumor signal is diluted by normal circulating molecules (derived from physiologic cells turnover and signaling or from clonal hematopoiesis), and the alterations found should be carefully checked to exclude false positive calls [12].

## 3. Liquid Biopsy as a Multi-Analyte Approach in Cancer Biology

### 3.1. Circulating DNA

Several analytes can be detected within liquid biopsy specimens and can be used to depict the biology of the tumor. Among these, ctDNA is currently the most widely used in clinical practice. In advanced solid tumors, ctDNA testing can complement or serve as an alternative to tissue-based molecular profiling to support treatment selection and to identify resistance mechanisms. ctDNA can also be directly used as a quantitative biomarker for patient stratification, tumor burden monitoring, and minimal residual disease (MRD) assessment, based on the clinical setting and assay sensitivity. Additionally, the analysis of ctDNA can be used to characterize and potentially stratify patients based on molecular alterations present (mutations, CNV, translocations) [6,8,9,12,13,14].

Globally, ctDNA analysis can be grouped into tumor-agnostic and tumor-informed approaches [15]. Tumor-agnostic strategies do not require prior knowledge of the patient’s tumor genotype and include methods such as low-pass whole-genome sequencing (to estimate the tumor fraction from broad copy number changes or from the study of the lengths of ctDNA fragments) [14,16,17,18,19], genome-wide methylation/epigenomic assays (for tumor fraction estimation, tumor methylation profiling, and cell-of-origin identification) [20,21,22,23], and comprehensive gene panel NGS assays for detecting all typical cancer-related mutations, copy number alterations, and rearrangements [24,25]. Examples of widely used clinical comprehensive genomic profiling tests include Guardant360 CDx (https://www.guardantcomplete.com, accessed on 20 February 2026) and FoundationOne Liquid CDx (https://www.foundationmedicine.com, accessed on 20 February 2026) [11].

Conversely tumor-informed approaches are based on previous knowledge of the alterations to be searched and can vary from single-target assays such as ddPCR [26,27] to several-target assays through small-target panel NGS, including commercially available tumor-informed MRD tests such as Signatera (https://www.natera.com, accessed on 20 February 2026) [6,28,29,30,31,32]. These tumor-informed approaches are typically more sensitive than tumor-agnostic methods [33], making them particularly suitable for MRD detection and longitudinal monitoring during the follow-up of patients. However, tissue requirements and turnaround time remain important limitations.

To improve the sensitivity and specificity of liquid biopsy tests, the simultaneous detection of more than one biomarker is highly desirable. However, limitations in sample availability, as well as time and cost constraints, remain major challenges.

To address these issues, novel multimodal technologies have recently been developed. These include the adaptation of Nanopore sequencers for ctDNA analysis—enabling the use of native long-read sequencing approaches even for short DNA targets—and the introduction of five-base chemistry for Illumina platforms [22,23,34]. This latter approach is able to preserve and detect cytosine methylation information without the need for bisulfite [34]. Together, these innovations make it possible to simultaneously detect both genomic alterations and epigenetic features using the same assay.

In addition to ctDNA, other sources of tumor information that are able to characterize and potentially stratify patients are extracellular vesicles and circulating tumor cells.

### 3.2. Circulating RNA

Among the circulating miRNAs, miR-371a-3p, in testicular germ cell tumors, represents the most clinically advanced example, with consistent diagnostic and monitoring performance across studies [35,36].

Several types of circulating RNAs can be detected in biological fluids [37], either as cell-free molecules or packaged within EVs or other circulating cellular components (e.g., CTCs, TEPs) [38,39,40]. Among these, microRNAs (miRNAs) have been extensively investigated as circulating predictive and prognostic biomarkers across multiple tumor types. miRNAs are remarkably stable in plasma and serum, as they are protected from degradation through encapsulation in vesicles or association with protein complexes, making them technically attractive for liquid biopsy applications.

Several circulating miRNA signatures have been reported across multiple cancer types, often in association with prognosis or response to different therapeutic regimens [41,42,43]. However, the majority of circulating miRNA biomarkers remain investigational and still require rigorous standardization and prospective multicenter validation before routine clinical implementation.

A notable exception is miR-371a-3p in testicular germ cell tumors, which represents one of the most clinically advanced examples of a miRNA-based liquid biopsy biomarker. Multiple studies have demonstrated the high sensitivity and specificity of miR-371a-3p for disease detection and monitoring, with performance that surpasses conventional serum tumor markers such as AFP, β-hCG, and LDH [35,36,44].

Exploratory studies have also investigated additional classes of short non-coding RNAs, including piRNAs, snoRNAs, and tRNA-derived fragments, as potential circulating biomarkers. Moreover, advances in transcriptome-wide profiling have enabled the detection of longer RNA species in plasma, such as coding RNAs, lncRNAs, circular RNAs and repetitive elements, which may provide complementary information on tumor biology [37,45,46,47,48,49].

Nevertheless, the clinical translation of circulating-RNA-based biomarkers remains challenged by substantial pre-analytical variability, low abundance, and the lack of harmonized protocols for RNA isolation, normalization, and data interpretation.

### 3.3. Circulating Protein

Proteomic profiling represents an additional and highly complementary dimension of liquid biopsy, enabling the detection of circulating tumor–associated proteins, inflammatory mediators, and microenvironment-derived signals. Traditionally, protein biomarkers in plasma have been assessed through targeted immunoassays such as ELISA or through mass-spectrometry-based workflows, which remain essential for unbiased discovery and for the characterization of proteins and post-translational modifications [50].

In clinical and translational settings, low- to mid-plex immunoassay platforms, including automated microfluidic ELISA systems, continue to provide robust and scalable quantification of selected proteins for validation and longitudinal monitoring [50]. However, recently, high-throughput affinity proteomics technologies have substantially expanded the depth and breadth of protein interrogation from minimal sample volumes. These latter platforms are based on proximity extension/ligation assays (e.g., Olink) or aptamer-based technologies (e.g., SOMAmer) and enable the simultaneous quantification of thousands of proteins, thus supporting large-scale biomarker discovery and multimodal integration with genomic and epigenomic liquid biopsy signals. These high-plex plasma proteomics have been applied for early detection [51,52,53], pre-diagnostic risk modeling in large cohorts, as well as therapy response profiling in cancers [54,55]. However, widespread clinical implementation will require further standardization, reproducibility assessment, and prospective validation.

### 3.4. Circulating Cells and Other Blood-Based Cellular Components

The analysis of CTCs can provide alternative information with respect to ctDNA. An important biomarker is the enumeration of CTCs. In breast cancer, large clinical studies support the CTC count as a robust prognostic biomarker in both early and metastatic disease, with growing evidence of the value of serial (repeated) enumeration in patient stratification [56].

Moreover, working with CTCs allows the isolation of viable tumor cells, providing a unique opportunity for ex vivo expansion, in vitro functional assays, and detailed molecular investigations at the single-cell level. Interestingly, CTC clusters (including heterotypic clusters with non-tumor cells, mainly from the immune compartment) have been identified as key mediators of metastatic dissemination [57]. This suggests that preserving cluster biology rather than dissociating samples provides additional biological insight and improves biomarker discovery.

Additional blood-based cellular components have been reported to act as carriers of tumor-derived information. Extracellular vesicles (e.g., exosomes/microvesicles) carry proteins and nucleic acids reflective of the tumor microenvironment and cell of origin and have been proposed as diagnostic/prognostic liquid biopsy biomarkers across disease stages [58,59,60]. However, EV biomarker translation remains strongly constrained by pre-analytical and analytical variability, making adherence to community standards (e.g., MISEV guidelines) central for reproducibility and clinical-grade validation [61].

Finally, TEPs have emerged as other potential sources of tumor-derived molecular information, with preliminary studies suggesting associations with prognosis and therapy response. Nonetheless, clinical evidence remains limited, and further studies are required [38,62].

## 4. Computational and Multimodal Integration

As liquid biopsy assays expand beyond single-variant detection, they increasingly generate high-dimensional and probabilistic data integrating somatic mutations with methylation profiles, fragmentomic features, and proteomic signals [14,63,64,65,66,67]. For example, cfDNA fragmentomic approaches such as DELFI leverage genome-wide fragmentation patterns and machine learning to detect and quantify tumor signals without prior tumor genotyping [14,63]. Machine learning frameworks can also improve classification and tissue-of-origin inference in methylation-based assays, as shown in large development and validation efforts such as the CCGA program for multi-cancer detection and cancer signal origin prediction [64,68].

Beyond these examples, a growing number of AI-based tools are being developed to integrate tissue and liquid biopsy molecular features with imaging, pathology, and clinical covariates, generating composite biomarkers that may outperform any single modality. However, such approaches also create new demands, including transparent validation, careful handling of batch effects, and avoidance of overfitting to narrow cohorts [69]. Ultimately, these computational advances will translate to the clinic only if supported by rigorous prospective evidence and harmonized analytical standards.

## 5. Challenges Facing the Clinical Translation of Liquid Biopsy

Several clinical applications of liquid biopsy are now established or rapidly consolidating. In advanced disease, ctDNA profiling can accelerate the identification of actionable alterations when tissue is insufficient or inaccessible, and it can enable the early detection of resistance mechanisms, supporting timely treatment adaptation.

A particularly active frontier is MRD, where ctDNA assays aim to detect residual tumor burden after surgery or definitive therapy. The conceptual appeal is strong: MRD could identify patients who benefit from treatment escalation while sparing others from overtreatment. However, this is also where the field must avoid overreach. MRD testing operates near the edge of detectability, with performance strongly influenced by tumor shedding biology, sampling timing, and assay design (tumor-informed vs. tumor-agnostic; mutation-based vs. methylation-based). Most importantly, prospective evidence is still required to demonstrate that ctDNA-guided strategies improve clinical outcomes, beyond simply correlating with recurrence risk [12].

Early detection and early multi-cancer detection comprise another high-impact but high-complexity domain. Here, the main biological limitation is signal scarcity: early lesions may shed little DNA, while non-malignant processes can generate overlapping background signals [68,70]. Equally critical are implementation challenges, including defining confirmatory pathways, minimization of downstream harms, and demonstration of population-level benefit.

Beyond biology, successful clinical translation depends on the harmonization of workflows and reporting standards. Pre-analytical variables (blood collection tubes, processing delays, storage conditions), assay characteristics (panel breadth, sequencing depth), and analytical definitions of positivity all shape sensitivity and interpretability. Moreover, cfDNA originates from multiple sources, and age-related clonal hematopoiesis can introduce tumor-unrelated somatic variants, complicating mutation attribution [71]. Robust pipelines, ideally supported by matched white blood cell sequencing or stringent filtering strategies, are therefore essential, particularly in therapy-guiding and MRD contexts.

Ultimately, aligning assay claims with realistic analytical constraints must proceed in parallel with health system considerations, including turnaround time, cost, reimbursement, and equitable access. As these challenges are addressed, liquid biopsy will increasingly shift from a complementary tool to a central component of precision oncology.

## Data Availability

No new data were created or analyzed in this study. Data sharing is not applicable to this article.

## Abbreviations

The following abbreviations are used in this manuscript:

MRD	minimal residual disease
TEPs	tumor-educated platelets
CTCs	circulating tumor cells
miRNAs	microRNAs
EVs	extracellular vesicles
ctDNA	circulating tumor DNA
